# COVID-19 infected cases in Canada: Short-term forecasting models

**DOI:** 10.1371/journal.pone.0270182

**Published:** 2022-09-22

**Authors:** Mo’tamad H. Bata, Rupp Carriveau, David S.-K. Ting, Matt Davison, Anneke R. Smit

**Affiliations:** 1 Turbulence and Energy Lab, Ed Lumley Centre for Engineering Innovation, University of Windsor, Windsor, Ontario, Canada; 2 Department of Statistical & Actuarial Sciences, Faculty of Science, Western University, London, Ontario, Canada; 3 Faculty of Law, University of Windsor, Windsor, Ontario, Canada; 4 Windsor Law Centre for Cities, Windsor, Ontario, Canada; Politecnico di Torino, ITALY

## Abstract

Governments have implemented different interventions and response models to combat the spread of COVID-19. The necessary intensity and frequency of control measures require us to project the number of infected cases. Three short-term forecasting models were proposed to predict the total number of infected cases in Canada for a number of days ahead. The proposed models were evaluated on how their performance degrades with increased forecast horizon, and improves with increased historical data by which to estimate them. For the data analyzed, our results show that 7 to 10 weeks of historical data points are enough to produce good fits for a two-weeks predictive model of infected case numbers with a NRMSE of 1% to 2%. The preferred model is an important quick-deployment tool to support data-informed short-term pandemic related decision-making at all levels of governance.

## 1. Introduction

Since December 2019, the severe acute respiratory syndrome coronavirus 2 (SARS-CoV-2) has infected more than forty million people across the globe. Despite the early warning and drastic large-scale measures that were immediately implemented by the Chinese government and the World Health Organization (WHO), COVID-19 spread outside the mainland of China in a matter of weeks [1]. The outbreak of COVID-19 evolved in three stages: locally in Wuhan, nationally in China, and worldwide in almost all countries [2].

Globally, countries implemented a plethora of pharmaceutical and non-pharmaceutical interventions to combat the ongoing COVID-19 crisis. Theses interventions affected people and the economy, leaving them with many undesirable uncertainties about the short to medium-term future. While such uncertainty is inevitable in a pandemic; leaders and policy makers must make use of predictions to chart steps forward for their constituents. To be useful, predictions should balance accuracy and speed of deployment.

Data analytics and prediction techniques can provide insightful feedback for past, present, and potential future pandemic response actions. Researchers have used these powerful tools in many pandemic applications [3–5]. Scarpino and Petri, 2019 [6] highlighted the importance of data analysis and prediction modeling in forecasting infectious disease outbreaks. Wheeler, 2019 [7] emphasized the value of machine learning prediction models in tracing disease outbreaks and informing public health organizations to avoid devastating pandemics. Chretien et al., 2015; Grassly and Fraser, 2008; and Colizza et al., 2007, [8–10] modeled infectious disease transmission risk in near real-time using data-driven models. While this is considered pre-pandemic and early stage modeling, other researchers analyzed current COVID-19 data and proposed prediction models that illustrate the criticality of this pandemic. For instance, Ng et al., 2020 [11] developed an age-structured agent-based model of the Canadian population that simulated the impact of current and projected levels of non-pharmaceutical public health interventions on COVID-19 transmission. Tuite et al., 2020 [12] used an age-structured mathematical model to assess the efficacy of implemented interventions on the transmission and peaks of COVID-19 in Ontario, Canada. Petropoulos and Makridakis, 2020 [13] presented simple time series models that predicted the continuation of COVID-19 spreading globally. Jia et al., 2020 [2] employed three mathematical prediction models to estimate the spreading patterns and peaks of COVID-19 in Wuhan and China at large. Fanelli and Piazza, 2020 [1] performed simple day-lag analysis and susceptible-infected-recovered-deaths (SIRD) analysis to forecast the evolution of COVID-19 in China, France, and Italy. Shao et al., 2020 [14] have also employed a standalone time-lag dynamic model and another fused with differential equations to simulate COVID-19 spreading scenarios in China, Singapore, and Diamond Princess Cruise Lines. A rich review on classification of machine learning forecasting models, criteria evaluation, and comparison of solution approaches related to COVID-19 were discussed by Rahimi et al., 2021 [15].

While no models are perfect, in engineering we must often forecast scenarios with incomplete understanding of the physics, inexact estimation of the parameters or the initial conditions, and considering unknown internal system response. Forecasts, even if unreliable, are still a necessary part of the governmental response to pandemic. COVID demands effective forecasting to help predict and manage hospital case loads, to guide public health measures including the availability of facilities and services, and to plan for the longer-term economic and social impacts of the pandemic.

Given the uneven spread of COVID by city and even neighbourhood (according to a variety of socio-economic determinants) [16], models which can be targeted to municipal- or even neighbourhood-based inquiry are of particular interest. However, in many cases, complex modelling is beyond the financial or logistical capacity of city governments or regional public health bodies.

This study focusses primarily on Canada as a case study. Throughout COVID-19, most Canadian modelling of predicted pandemic spread has taken place at the provincial or national level. Yet in a country as physically vast and geographically and demographically diverse as Canada, patterns of pandemic spread have varied widely between municipalities. Even school closures have in some provinces at least been addressed on a region-by-region basis. Municipalities in Canada have decision-making powers which during COVID-19 have included regulating transit service and store and restaurant closures or restrictions, and the implementation and enforcement of physical distancing and mask bylaws. Three potential models are tested. One is ideal for use by municipalities in its simplicity and cost-effectiveness. Given this, it offers an accurate and practical option for cities to assist in tailoring their public health measures, and their predictions to the local community.

## 2. Forecasts

Researchers and developers employ short-, medium-, and long-term forecasting models that learn past trends to optimize a future process. Forecasting methods can be classified into qualitative and quantitative. Qualitative techniques are subjective and based on experts’ judgments and are mostly useful when past data is not available or reliable. Quantitative techniques, on the other hand, are data-driven and rely on past trends for future forecasts. In this study, short-term quantitative forecasting models are developed given the assumption that data are reliable. The usability of short-term forecasting models stems from their accuracy and robustness which is driven by many parameters such as model structure, input data type, forecasting horizon, and temporal resolution of the fed data [17]. Here, the focus is on the forecasting model results interpretation as a function of forecasting horizon. While structure and calibration play a main role in a model’s performance and accuracy, variations and uncertainties in the input data should be investigated first. For COVID-19, a wide spectrum of variations and uncertainties could be argued.

First, the unquantifiable uncertainty that hails from limitations in estimating the reliability and accuracy of input data. Available input data have been shaped by wide differences in location, population density, economic activities, individuals’ health, individuals’ acceptance and reaction to the outbreak, pharmaceutical non-pharmaceutical interventions, politics and policies, testing density, testing strategy, data collection and reporting, and many more–variables that are not quantified here. The data on the number of COVID-19 deaths could be among the more reliable variables to forecast than number of cases, tests, or recoveries; however, it still suffers from additional uncertainties and biases such as: possible death caused from pre-existing conditions, and possible incentivised underreporting to avoid panic-mongering and economic fallout.

Second, uncertainties stem from the uncollected data of subclinical and untested-unidentified cases. Forecasting models use available data on number of infected, death, and recovered cases while subclinical and untested-unidentified cases contribute to the spreading of the disease. In the COVID-19 pandemic, the subclinical and untested-unidentified category includes clinically undiscovered cases (i.e., mild-symptomatic and asymptomatic), and individuals who refuse hospitalization for ideological and conspiratorial backgrounds. Such backgrounds add another spectrum of variations (e.g., public awareness, education level, and other socio-economic factors) to the forecasting model.

Despite the abovementioned uncertainties and variations in the model input data, imperfect forecasts can still be useful. The output could be used as a guideline for government decision making. At all levels, to guide provincial decisions on stay-at-home orders, school and business closures, and restrictions on social gatherings, for example, or municipal-level decisions on availability of transit and other services, mask and physical distancing by-laws, the closure of city-run amenities including community centres, arenas, and skating rink. From a governance perspective, modelling is only one tool to guide decision-making during pandemic, which can never be divorced from the political, economic and social context in which it is taking place [18, 19], but it is an important tool. At a municipal level, modelling may also be targeted to focus exclusively on a particular neighbourhood or part of the city, to take into account demographic differences and equity considerations in various possible responses.

A simple trend analysis model (denoted Trendline), a Support Vector Machine (SVM) regression model, and a Gaussian Processing Regression (GPR) model are employed to forecast the total number of COVID-19 infected cases in Canada two weeks into the future. As mentioned before, the focus here is on the model’s output and interpretation than the model’s development. Horizons of 5, 10, 15, 20, 25, and 30 days were selected to meet the model applicability and usability [20, 21]. A shorter horizon forecast may provide more accurate output; however, the forecast loses part of its applicability. On the other hand, a longer horizon forecast would be more useful, but it loses part of its accuracy. Therefore, the trade-off between usability and accuracy is an optimization process which, in this paper’s short-term application has been investigated.

## 3. Forecasting methodology and models

**Fig 1:**
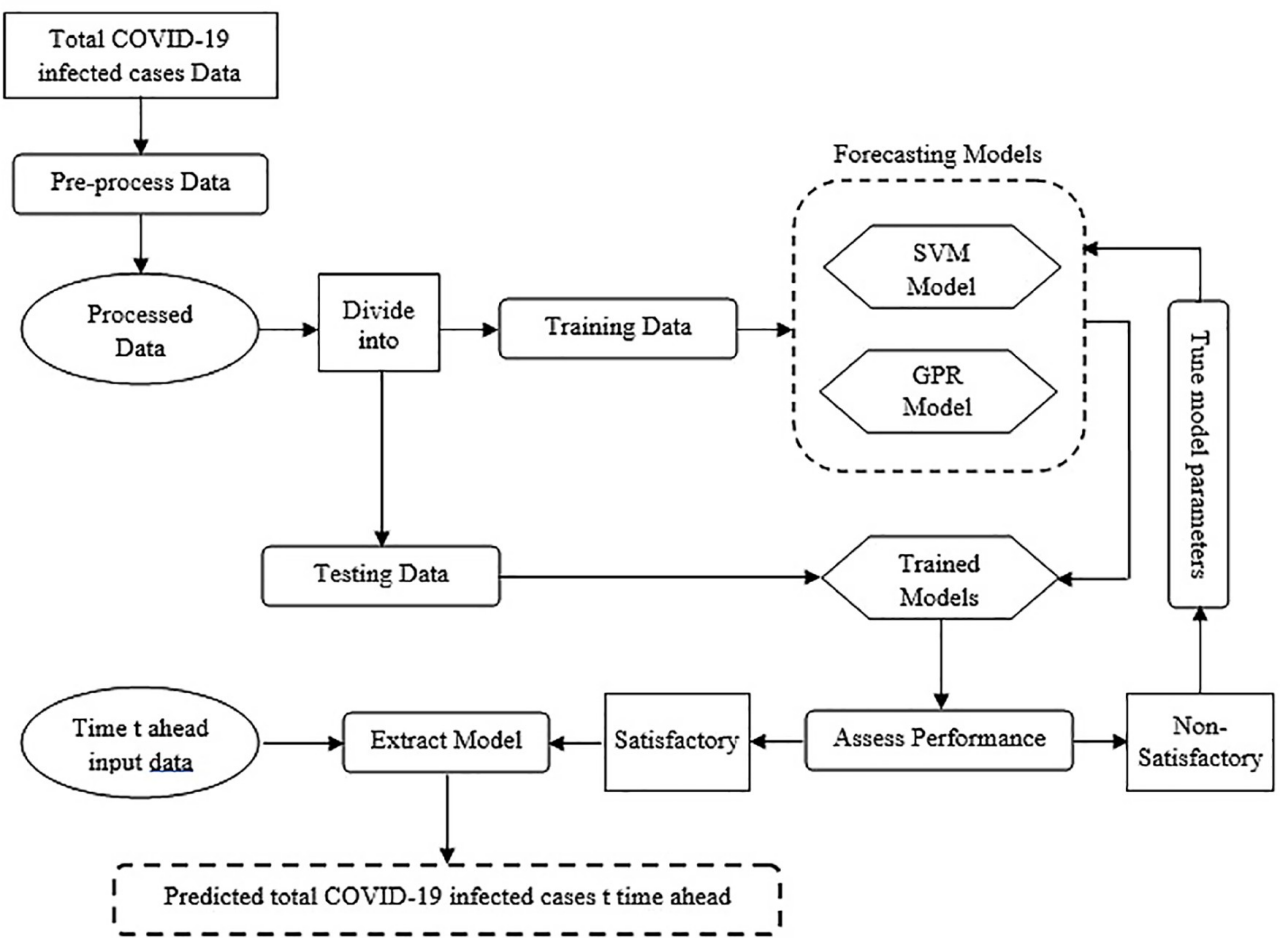
Forecasting methodology flowchart.

This section describes the forecasting methodology, the forecasting approach, the forecasting models, and the model performance assessment approach. Fig 1 depicts a flowchart of the forecasting methodology adopted in this work. The forecasting process starts with data collection for the studied area. The data at this point is still unprocessed. Here, this raw data is referred to as pre-processed data. Pre-processed contains erroneous data (e.g., negative number of infected cases), noisy data (e.g., untrue number of infected cases), and/or missing data. The pre-processing phase is to ensure the integrity of the through cleaning and imputation, where it becomes processed data ready for use. Then, processed data is divided into two sets where time order was kept as in the original timeseries: 1) training set, 75% of the processed dataset, which is fed to two separate models, SVM model and GPR model. 2) testing set,25% of the processed data, is used for testing and validating the models after they are trained. This data division configuration was used based on the guidelines of Hunter et al., 2012 [22]. Models’ performance is measured at this point. If model’s prediction performance is satisfactory, then the model can be extracted and deployed and can be fed with the time ahead input data to predict the target, total COVID-19 infected cases. Ordinarily, models undergo a number of calibrations and tunings before their prediction performance passes a satisfactory threshold. An example of such calibrations is selecting a different Kernel.

An 11^th^ generation core i7 with 16 gigabytes installed RAM and x64 based processor machine was used to develop, train, tune, and validate proposed models. Microsoft Excel spreadsheets and data analysis function were used to investigate the simple trend analysis model. And GUI Classification Learner Application kit in MATLAB R2020a was deployed to generate, train, and tune the SVM and GPR models. Models source codes and tuned parameters could be extracted and exported through the same kit, the Classification Learner Application.

### 3.1 Simple trend analysis model

Statistically, trend analysis is widely used in finding a distinguished pattern or behaviour in time series data. Trend analysis models are relatively easy to build and deploy due to their simple structure. However, their robustness might be lower than other sophisticated techniques. This technique can be as simple as performing linear regression on a dataset with apparent linearity or very low nonlinearity. Such analysis offer performing non-parametric methods (e.g., Mann-Kendall test) on datasets with nonlinear patterns. In this work, trend analyses were carried out by fitting polynomials (see Eq 1) with different degrees (n) to the dataset and measuring their coefficient of determination (R^2^).


$$a_{n}Y^{n}+a_{n-1}Y^{n-1}+\cdots +a_{2}Y^{2}+a_{1}Y+a_{0}$$ (1)

$$R^{2}=1-\left(\frac{\sum_{i}^{n}\left(Y_{i}-Y_{P}\right)}{\sum_{i}^{n}(Y_{i}-Ȳ)}\right)$$ (2)

Where α_0_, …, α_n_ are constants, Y is the indeterminate target, Y_i_ is the observed indeterminate data point, Y_p_ is the predicted indeterminate data point, and Ȳ is the indeterminate dataset mean.

### 3.2 Support Vector Machine model (SVM)

Introduced first by Boser et al., 1992 [23], SVM has since been studied, developed, optimized, and intensively applied to a wide range of problems [24]. The process of developing SVM generates the general class of algorithms called Kernel [25]. SVM is simply a supervised linear classifier that separates a dataset into classes, that have maximum margin between them to achieve minimum generalization error, with a hyperplane. The data points that create the hyperplane are called the support vectors. For SVM, optimal hyperplanes are found through a repetitive process of guessing [26]. The mathematical formulation of SVM is explained thoroughly in many rich literature references [24, 27, 28].

SVM models adopt the structural risk minimization principle, which performs by minimizing the generalization error instead of the training error. This makes SVM more robust than many other forecasting techniques. SVM maps data points nonlinearly onto a high dimensional feature space and then applies linear regression in this space. The regression function used has the following form:

f(x)=ωɸ(X)+b (3)

where, ɸ is the feature (i.e., the kernel function here) for which data are nonlinearly mapped into space X. The coefficients ω and b are estimated by minimizing the risk function, R(C):

$$Minimize\;R(C)=C\frac{1}{N}\sum_{i=1}^{N}L\varepsilon \;(di,Yi)+\frac{1}{2}{\left\|\omega \right\|}^{2}$$ (4)

$$Subject\;to:\;L\varepsilon \;(di,Yi)=\left\{\begin{array}{l} \;|d-Y|-\varepsilon ,|d-Y|\geq \varepsilon \\ 0\;,|d-Y|<\varepsilon \end{array}\right\}$$ (5)

where, C is the regularized constant that determines the trade-off between the empirical error and the regularization term, N is the total number of data points, Lε (d,y) is the ε-insensitive loss function, ε is a stipulated parameter that determines the threshold of the error penalty, and di is the actual total COVID-19 infected cases value at point i.

In order to obtain a direct solution without the challenge of actual data mapping (i.e., feature ɸ), kernel functions are applied [29]. In an application where the dataset has a degree of nonlinearity, like this application here, polynomial kernel functions are sought efficient and useful compared to linear and radial basis functions (RBF). Polynomial kernel functions can be represented as:

$$K(Xi,Xj)={\left(Xi∙Xj+C\right)}^{d}$$ (6)

Where d is the degree of the kernel function (e.g., a quadratic kernel function has a degree d equal to 2). Now, if we introduce Lagrange multipliers, αi, αi* (αiαi* = 0, αi, αi* ≥ 0) and combine with the polynomial kernel function, Eq 3 becomes:

$$f(x)=f(X,\alpha i,{\alpha i}^{*})=\sum_{i=1}^{N}(\alpha i-{\alpha i}^{*})∙K(X,Xi)+b$$ (7)

### 3.3 Gaussian Processing Regression model (GPR)

GPR is a probabilistic nonparametric kernel-based Bayesian approach to regression that assumes a prior probability distribution over a latent function. A GPR model explains the response by introducing latent variables, f (x_i_), I = 1, 2, 3, …, n, from a Gaussian Process (GP) and an explicit basis function [30]. A GPR model predicts a future target value (e.g., Y_new_) given the new input vector X_new_, and the training data of (X_i_, Y_i_) vectors. This relationship between the input vector (X) and target (Y) in GPR model is formulated as:

Yi=f(Xi)+ε (8)

Where ε here is an independent additive white noise that is subjected to a Gaussian distribution with zero mean and a variance of σ^2^, that is ε ~ N(0, σ^2^). Andersson and Sotiropoulos, 2015 [31] determined f(x) and any other unobserved pair (f*, x*) as:

$$\left[\begin{array}{c} f\\ f^{*} \end{array}\right]∼N_{n+1}\left(0,[\begin{array}{c} K(X,X){+\sigma }^{2}IK(X,x^{*})\\ K(x^{*},X)\;K(x^{*},x^{*}) \end{array}]\right)$$ (9)

where K (X, X) is a n x n covariance matrix between all the samples in the training data, and I denotes an n dimensional identity matrix. This covariance function expresses the similarities in Gaussian processes [30]. This covariance function can be defined by various kernel functions. In terms of kernel parameters in vector θ, it is possible to express the covariance function as K (X, X│θ). For various types of kernel functions, the kernel parameters are dependent on the signal standard deviation σ_F_, and the characteristic length scale σ_L_. Both σ_F_ and σ_L_ have to be greater than zero, which is guaranteed by the unconstrained parameterization vector θ, such that θ_1_ = log σ_F_, θ_2_ = log σ_L_.

Four kernel functions were applied in this study, namely: the exponential, the squared exponential, the Matern 5/2, and the rational quadratic, presented in Eqs 10 to 13, respectively.


$$K(Xi,Xj|\theta )=\sigma_{F}^{2}\;\exp\left(-\frac{r}{\sigma_{L}}\right)$$ (10)

$$K(Xi,Xj|\theta )=\sigma_{F}^{2}\;\exp\;[-\frac{1}{2}\frac{{\left(Xi-Xj\right)}^{T}(Xi-Xj)}{\sigma_{L}^{2}}]$$ (11)

$$K(Xi,Xj|\theta )=\sigma_{F}^{2}\;\left(1+\frac{\sqrt{5}r}{\sigma_{L}}+\frac{5r^{2}}{3\sigma_{L}^{2}}\right)\;\exp\;\left(-\frac{\sqrt{5}r}{\sigma_{L}}\right)$$ (12)

$$K(Xi,Xj|\theta )=\sigma_{F}^{2}\;{\left(1+\frac{r^{2}}{2\alpha \sigma_{L}^{2}}\right)}^{-\alpha }$$ (13)

Where α is a positive-valued scale-mixture parameter, and r is the Euclidean distance between X_i_ and X_j_ and can be calculated using Eq 14.


$$r=\sqrt{{\left(Xi-Xj\right)}^{T}(Xi-Xj)}$$ (14)

### 3.4 Model performance

The three forecasting (i.e. Trendline, SVM, and GPR) were deployed to forecast the total number of infected cases in Canada during the months of April, May, June, and July using daily timesteps and an incremental horizon of 5 days. The performance of the models was measured based on the deviation of the predicted total number of infected cases from the actual total number of infected cases. The forecasting error was measured for all forecast points (i.e. overall performance), and for only the last 5 points of the forecast. The forecasting error was measured using: (1) the Mean Absolute Percentage Error (MAPE) and (2) the Root Mean Squared Error (RMSE). The Normalized Root Mean Squared Error (NRMSE) was also calculated and shown along with MAPE in Table 1 to account for the variation of the means of datasets used in forecasting the total number of infected cases. Eqs 15, 16, and 17 represent the MAPE, RMSE, and NRMSE, respectively.

$$MAPE=\frac{1}{n}\sum_{i=1}^{n}\left|\frac{Yi-Ŷi}{Yi}\right|$$ (15)

$$RMSE=\sqrt{\frac{1}{n}\sum_{i=1}^{n}({\hat{Y_{i}}}^{2}-Y_{i}^{2})}$$ (16)

$$NRMSE=\frac{RMSE}{\bar{Y_{i}}}$$ (17)

Where,

n: is the number of data points

Yi: is the forecasted total number of infected cases

Ŷi: is the actual total number of infected cases

$\bar{Y_{i}}$: is the data set mean

**Table 1:** Models configuration and overall performance.

Round	Forecast span	Number of input data indices	NRMSE (%)			MAPE (%)		
			Trendline	SVM	GPR	Trendline	SVM	GPR
Round 1	April 06 –April 19	26	11.4	9.5	8.5	11.7	9.5	6.7
Round 2	April 20 –May 03	40	10.7	14.4	0.9	10.1	11.3	0.5
Round 3	May 04 –May 17	114	12.1	4.4	1.1	8.9	3.4	1.0
Round 4	May 18 –May 31	128	1.5	2.1	2.2	1.3	2.0	1.8
Round 5	June 01 –June 14	70	0.9	1.2	0.9	0.6	1.0	0.6
Round 6	June 15 –June 28	63	1.9	1.8	1.4	1.6	1.7	1.1
Round 7	June 29 –July 12	49	1.7	0.5	0.7	1.5	0.4	0.5
Round 8	July 13 –July 26	49	1.7	0.9	0.6	1.3	0.8	0.6

## 4. Results and discussion

Data of daily COVID-19 infected cases were collected from Our World in Data (OWID), University of Oxford [32]. Analysed data spans the period from February 11, 2020 until July 20, 2020 for Canada. Here, we assume the reliability of data collected and updated by OWID from the European Centre for Disease Prevention and Control (ECDC). Although this study is to propose cost-effective, simply deployable short-term forecasting models at the municipality level, Canadian COVID-19 infected cases data was employed for this reason. This is because at the time of this study, national-level datasets were more acceccable and reliable compared to municipal-level datasets.

### 4.1 Overall performance

**Fig 2:**
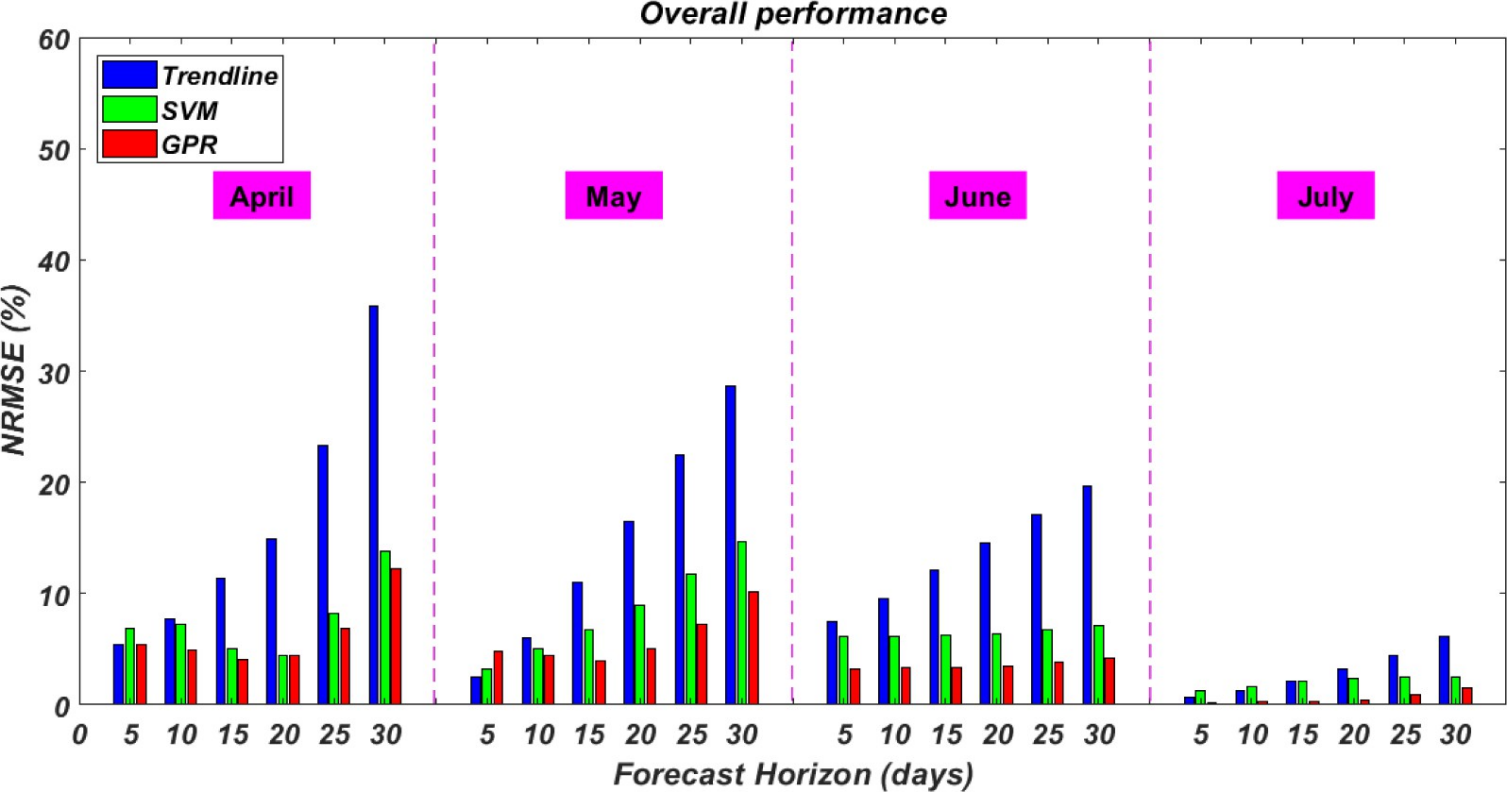
Models overall performance.

The overall performance results for the three proposed models forecasting 5, 10, 15, 20, 25, and 30 days ahead are presented in Fig 2. It can be seen that the forecast in all time horizons have a better performance when GPR and SVM models are deployed compared to the simple Trendline model. On average, the NRMSE for Trendline was 5% to 25% higher compared to SVM and GPR models. This is expected, as the two machine learning models (i.e. SVM and GPR) have a better prediction ability compared to the simple traditional trend analysis. Also, it can be seen that as the forecasting horizon increases, the NRMSE for Trendline increases in a steeper fashion compared to SVM and GPR models. This is important in the application of forecasting as it is an indicator of model robustness. Another observation that can be drawn from Fig 1 is regarding the forecasting horizon. Forecasts with longer forecasting horizons have a higher NRMSE. This is also expected, especially in forecasting the total number of infected cases, because changes in policies, politics, human behaviours, and other socio-economic parameters happen within days or weeks. The last observation in the models’ overall performance has to do with the input data. The three proposed models had an improvement in their forecasting performance moving from April through July. This improvement in the models’ forecasting performance is here thought to be driven by the input data. It is plausible to say that input data for the later months had more information and offered better explanation of the pandemic trends.

### 4.2 Last five points performance

**Fig 3:**
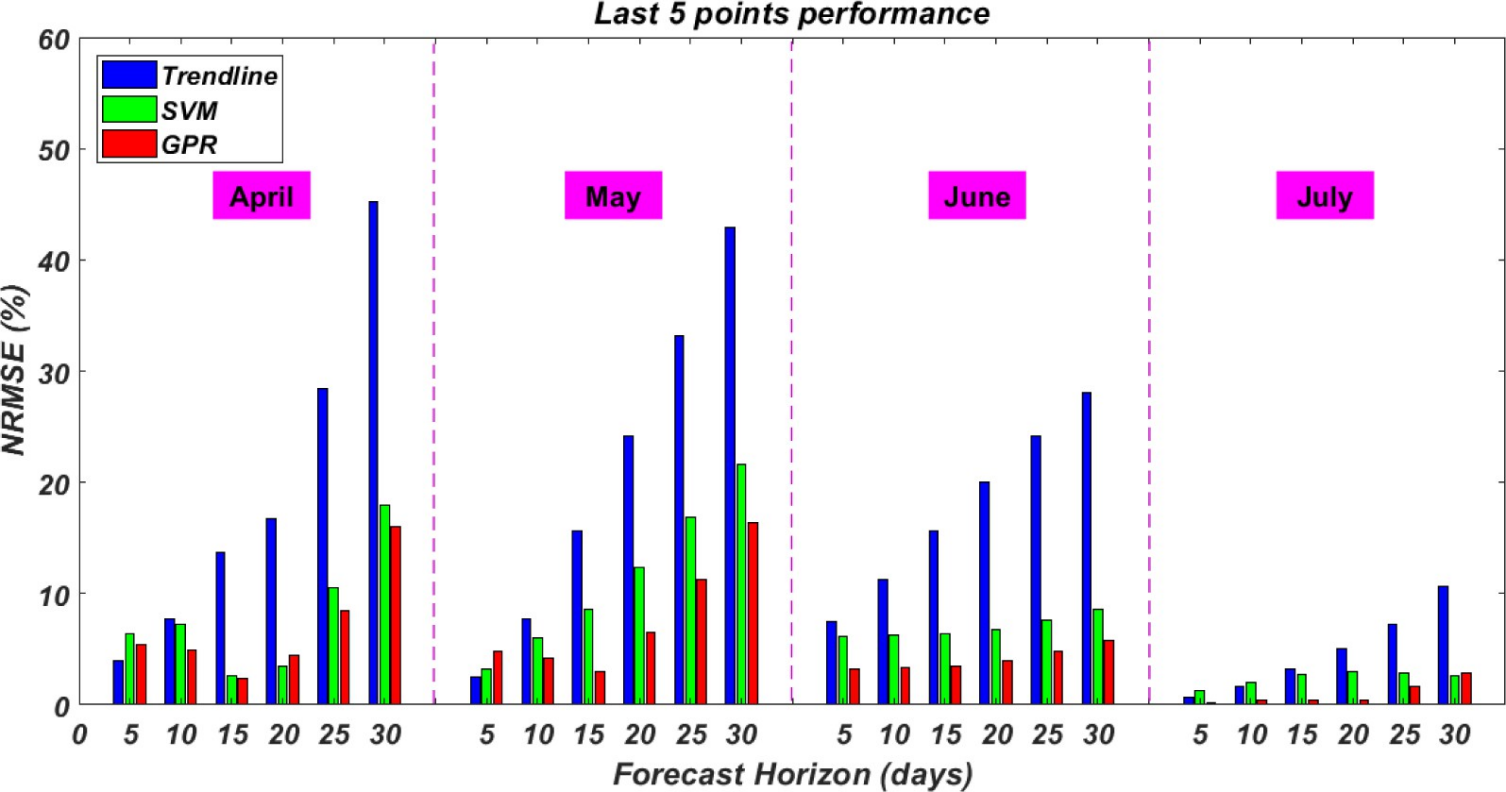
Models performance measured over the last 5 points of the forecast.

In this section, a different metric is presented to evaluate the models’ performance. some decisions and applications regarding COVID-19 pandemic concern with the total number of infected cases at the end of the forecasting horizon rather than the pattern. Therefore, instead of including all points in calculating the deviation of the predicted total number of infected cases from the actual, only the last five points are considered. The results of this metric are shown in Fig 3. The patterns of error show in Fig 2 are also present in Fig 3, albeit at greater magnitude in the latter. While no change is seen in the 5 days forecasting horizon, longer horizons see larger changes in NRMSE. This is anticipated as uncertainties in the forecast increase with longer horizons. Again, it is important to highlight the trade-off between a forecasting model’s accuracy and usability. In this paper, a mid-point in the forecasting horizon of two weeks is considered reasonable in the estimation of total number of infected cases. In the next section, the three models are deployed to forecast two weeks ahead.

### 4.3 Models application

#### Rounds 1 and 2

**Fig 4:**
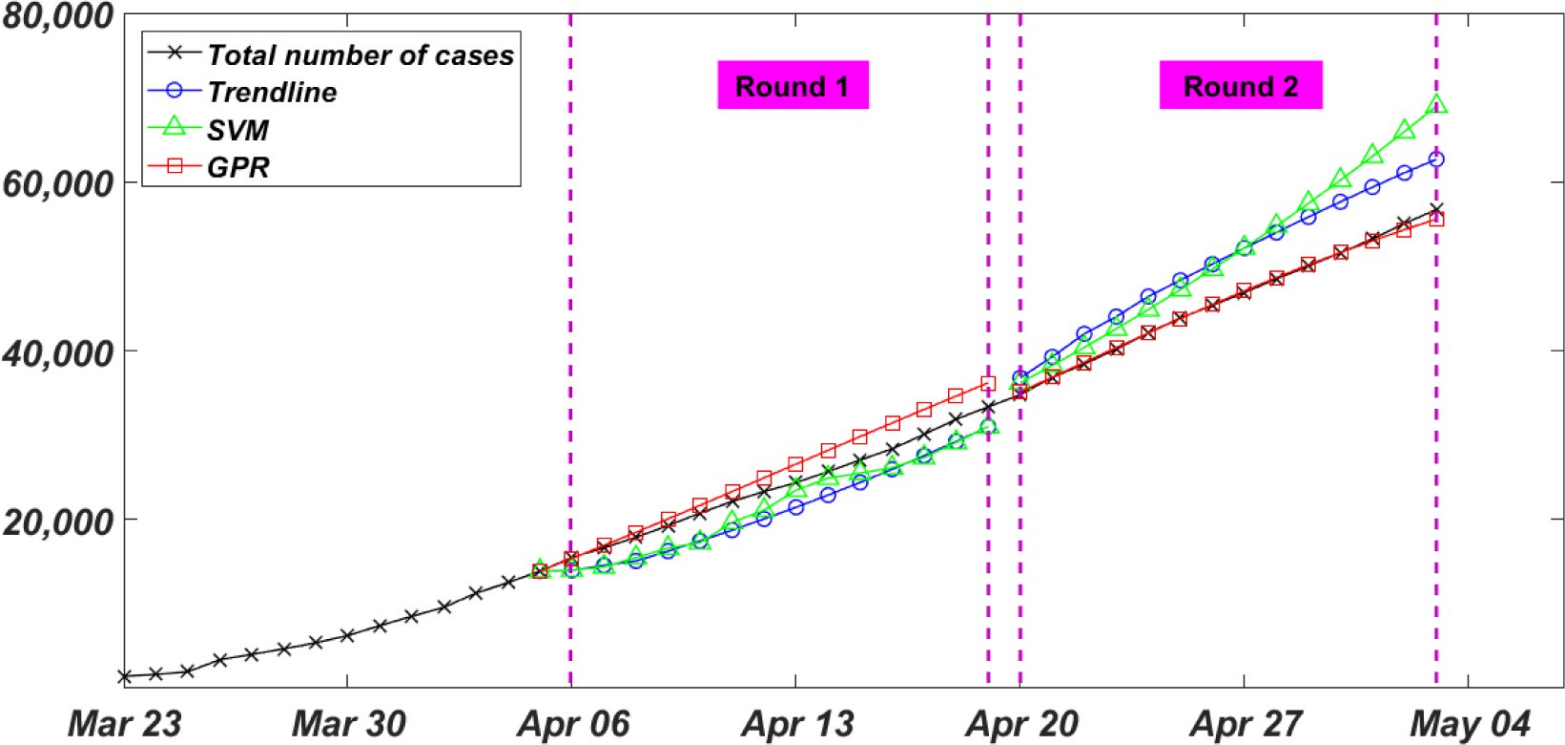
Rounds 1 and 2 of the bi-weekly total number of infected COVID-19 cases in Canada.

The first and second forecasts (i.e. Round 1 and Round 2) were performed on April 05 and April 19, respectively. Round 1 forecast was for the total number of infected cases in Canada between April 06 and April 19 as shown in Fig 4. A total of 26 data points, since 100 infected cases were reported, were used to fit, and train the forecasting models. In this round, the Trendline model was fitted using a third degree polynomial. Other degrees of polynomials and other trend functions were fitted too, however, the third degree polynomial had the least RMSE and seemed to explain the curve growth better than the other functions. In the same fashion, different SVM regression models were trained, and the cubic SVM regression model had the least RMSE. Also, for the GPR model, amongst all the Kernel functions deployed to train the data, the Matern 5/2 Kernel performed best with the least RMSE.

On April 05, when Round 1 forecast was performed, the total number of infected cases was 13882. Two weeks later, on April 19, the total number of infected cases increased to 33341. This number was forecasted to be 30989, 30999, and 36180 by Trendline, SVM, and GPR models, respectively. The calculated NRMSE for this round, shown in Table 1, was 11.4%, 9.5%, and 8.5% for the Trendline, SVM, and GPR models, respectively. Within these two weeks, the Total number of infected cases dramatically increased by approximately 140%. This, accompanied by a small number of input data used in fitting the models, was identified as a source of uncertainty and error in the forecast in this round.

Round 2 forecast was for the period between April 20 and May 03 as shown in Fig 4. A total of 40 data points, since 100 infected cases were reported, were used to fit, and train the forecasting models. In Round 2, the same technique of fitting and modeling was followed. A cubic polynomial Trendline model, a cubic SVM regression model, and a Matern 5/2 Kernel GPR model had the least RMSE, and therefore were deployed to forecast the total number of cases two weeks ahead.

On April 19, the Round 2 forecast was performed, the total number of infected cases was 33341. Two weeks later, on May 03, the total number of infected cases increased to 56714. This number was forecasted to be 62676, 68985, and 55581 by Trendline, SVM, and GPR models, respectively. The calculated NRMSE for this round, shown in Table 1, was 10.7%, 14.4%, and 0.9% for the Trendline, SVM, and GPR models, respectively. The Matern 5/2 covariance function was able to decrease the foresting error by about 8% compared to previous round. It is worth mentioning that both Trendline and SVM models were still following a cubic explanation of the growth in the total number of infected cases. This is likely the reason for a high forecasting error and an overestimation of six to twelve thousand cases. This improved when Rounds 3 and 4 were carried out.

#### Rounds 3 and 4

**Fig 5:**
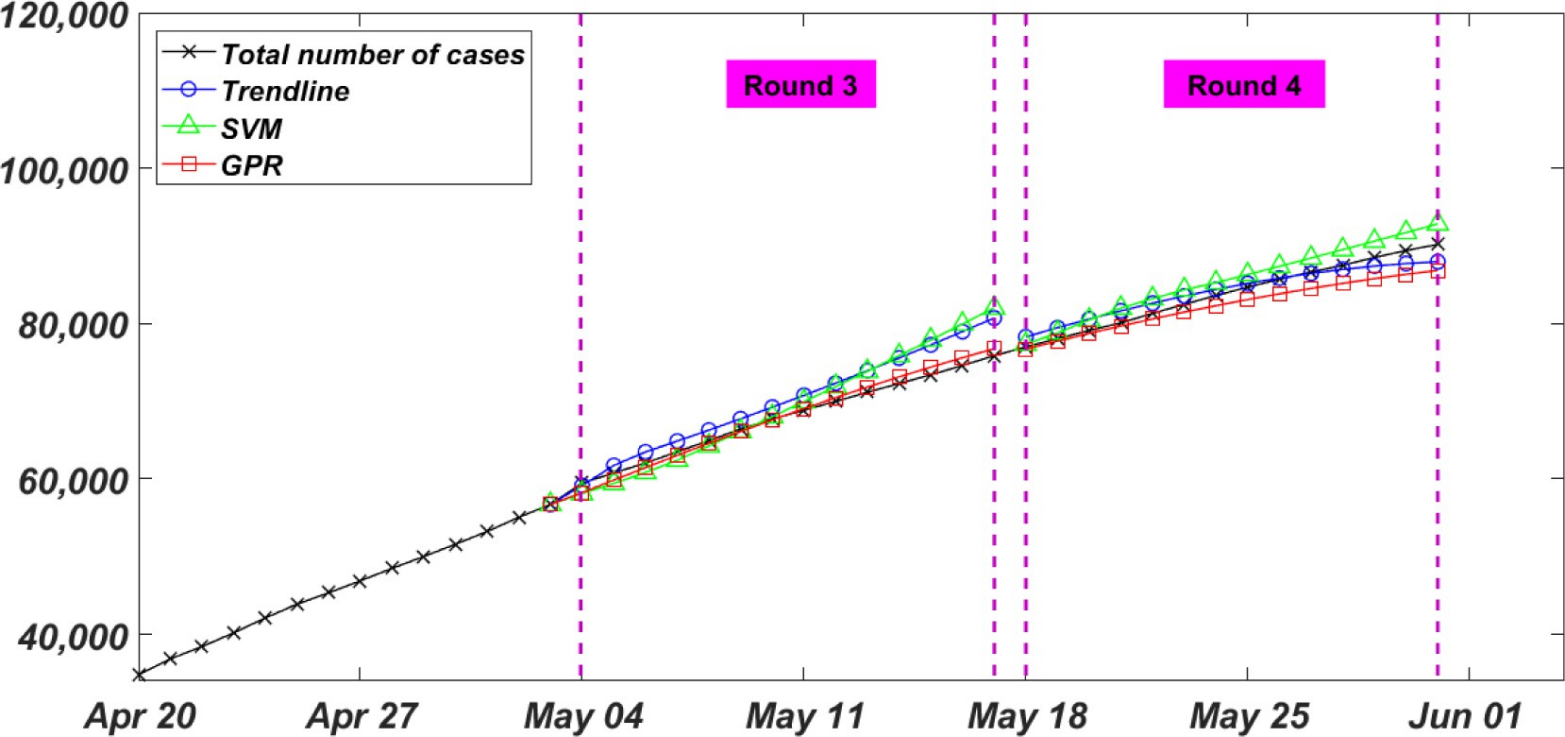
Rounds 3 and 4 of the bi-weekly total number of infected COVID-19 cases in Canada.

The third and fourth forecasts (i.e. Round 3 and Round 4) were performed on May 03 and May 17, respectively. In Round 3, forecasting models were deployed to predict the total number of infected cases for the two weeks between May 04 and May 17 as shown in Fig 5. In this round and as mentioned above, a cubic extrapolation function was observed to overestimate the total number of infected cases in Canada. Therefore, the simple trend analyses were performed using a quadratic polynomial to fit a total input of 114 data points. A quadratic, instead of cubic, SVM regression model, and a Matern 5/2 kernel GPR model were also trained using the total of 114 data points. At the end of the two weeks forecast, the actual total number of infected cases of 75853 was predicted to be 81161, 82005, and 76762 using the Trendline, SVM, and GPR models, respectively. The calculated NRMSE for this round, shown in Table 1, was 4.2%, 4.4%, and 1.1% for the Trendline, SVM, and GPR models, respectively. Both the Trendline and SVM models executed the forecast with less NRMSE compared to the previous round, while the GPR model achieved the least NRMSE and the most accurate prediction amongst proposed models.

On May 17, the round 4 forecast was executed for the two weeks between May 18 and May 31 as shown in Fig 5. A total of 128 data points were used as an input for the forecasting models. The predicted total number of infected cases at the end of this round was 87953, 92827, and 86841 by Trendline, SVM, and GPR, respectively, compared to 90179 as the actual total number of infected cases. Similar to the previous round, the Trendline model was fitted using a quadratic polynomial, the SVM model was trained using a quadratic function, and the GPR was trained using the Matern 5/2 kernel function. The calculated NRMSE for this round, shown in Table 1, was 1.5%, 2.1%, and 2.2% for the Trendline, SVM, and GPR models, respectively. A significant drop in forecasting error for Trendline and SVM models was observed during this round. This drop likely stemmed from a steady daily change in the total number of infected cases. Within these two weeks the new daily infected cases started to drop to an average of 1000 cases with a deviation of 200 cases. A smaller deviation from the mean within these two weeks minimized the effect of outliers and high deviated data points, and therefore less error and uncertainty in the models’ performance. That said, input data with continuous change in trend and deviation may result in a higher uncertainty in the models’ ability to predict. This notion was applied when using shorter spans of input data in rounds 5, 6, 7, and 8 to fit and train models.

#### Rounds 5 and 6

**Fig 6:**
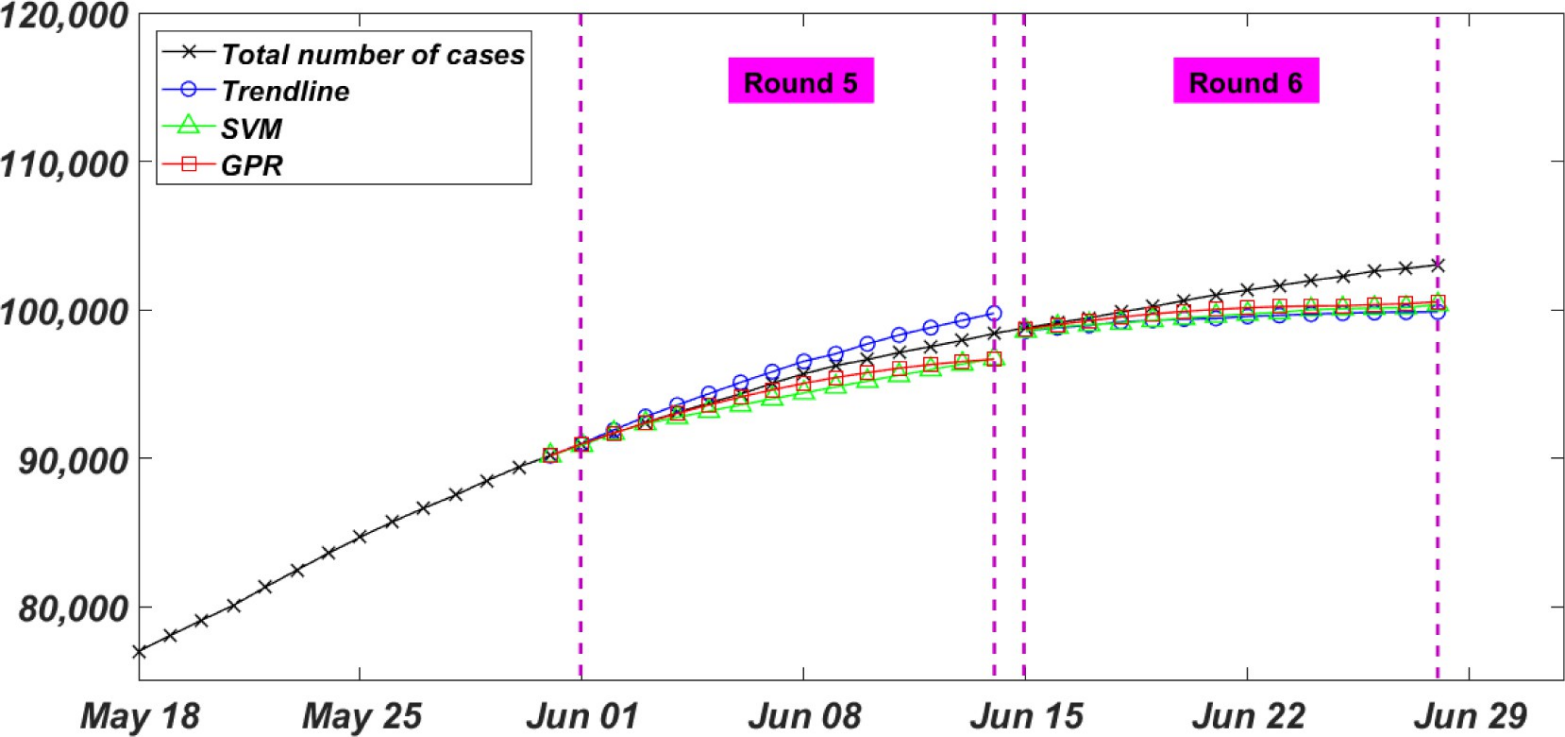
Rounds 5 and 6 of the bi-weekly total number of infected COVID-19 cases in Canada.

The fifth and sixth forecasts (i.e. Round 5 and Round 6) were performed on May 31 and June 14, respectively. In Round 5, forecasting models were deployed to predict the total number of infected cases for the two weeks between June 01 and June 14 as presented in Fig 6. A total of 70 data points (10 previous weeks) was found to achieve the best fits with the least RMSE. This number of data points was optimized by investigating different fits with input data that have different span length. A quadruple polynomial, a medium Gaussian Kernel, and a Matern 5/2 Kernel were used to fit the Trendline, SVM, and GPR models, respectively. At the end of this round, the actual total number of infected cases of 98399 was predicted to be 96666, 96713, and 97281 by Trendline, SVM, and GPR, respectively. Models in this round achieved a very low NRMSE of, shown in Table 1, 0.9%, 1.2%, and 0.8% for the Trendline, SVM, and GPR models, respectively. This increase in models’ predictivity was due: 1) less variation in the daily change of number of infected cases, and 2) optimizing the number of previous input data points that lead to the fitted models with the least RMSE.

In Round 6, forecasting models were deployed to predict the total number of infected cases for the two weeks between June 15 and June 29 as Fig 6 depicts. A total of 63 data points (9 previous weeks) was found to achieve the best fits with the least RMSE. Similar to the previous round, the number of data points was optimized by trial and error for different fits with input data that have different span length. A quadruple polynomial, a cubic SVM, and a Matern 5/2 Kernel were used to fit the Trendline, SVM, and GPR models, respectively. At the end of this round, the actual total number of infected cases of 103021 was underestimated to be 99852, 100352, and 100534 by Trendline, SVM, and GPR, respectively. Models in this round achieved a low NRMSE of, shown in Table 1, 1.9%, 1.8%, and 1.4% for the Trendline, SVM, and GPR models, respectively. Models in this round predicted a steep decrease in the new daily infected cases. On average, the three predictive models underestimated the total number of infected cases by 3000. That is explained by following the previous two weeks trend of reduction in the new daily infected cases.

#### Rounds 7 and 8

**Fig 7:**
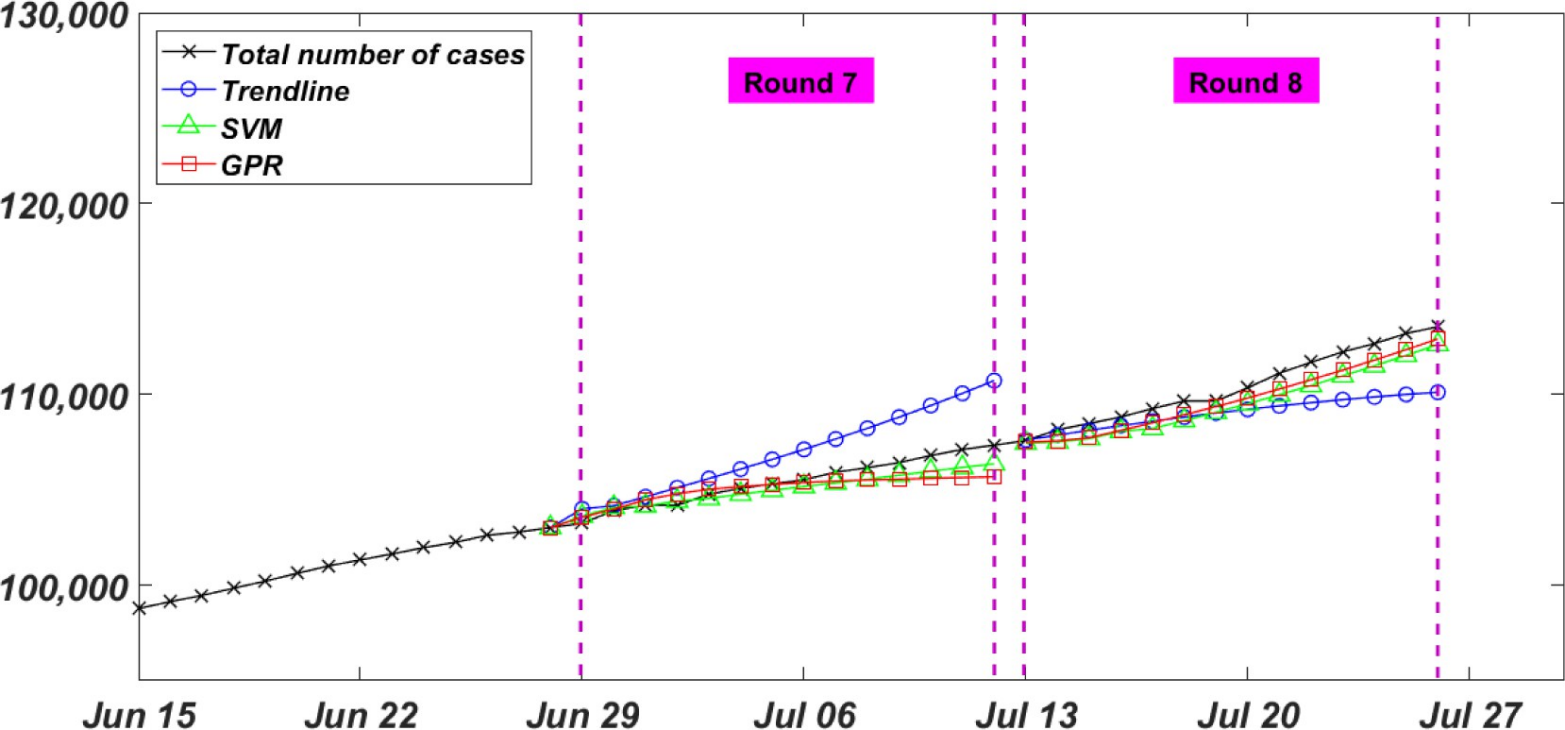
Rounds 7 and 8 of the bi-weekly total number of infected COVID-19 cases in Canada.

The last two rounds (i.e. Round 7 and Round 8) in this paper were executed on June 28 and July 12, to forecast the total number of infected cases, shown if Fig 7, between June 29 and July 12, and July 13 and July 26, respectively. Models in both rounds were fed with 49 (7 previous weeks) input data points. In Round 7, the Trendline, SVM, and GPR models were fitted and trained with a cubic polynomial, a quadratic SVM function, and a Matern 5/2 Kernel, respectively. While a quadruple polynomial, a cubic SVM function, and a Matern 5/2 Kernel were used in Round 8, respectively. At the end of Round 7, the actual total number of infected cases of 107335 was predicted to be 110719, 106357, and 105679 by Trendline, SVM, and GPR, respectively. Models in this round, again, achieved a very low NRMSE of, shown in Table 1, 1.7%, 0.5%, and 0.7% for the Trendline, SVM, and GPR models, respectively. Analogously, in Round 8, the actual total number of infected cases of 113543 was predicted to be 110110, 112610, and 112910 with a NRMSE of, shown in Table 1, 1.7%, 0.9%, and 0.7% for the Trendline, SVM, and GPR models, respectively.

### 4.4 Models uncertainty

**Fig 8:**
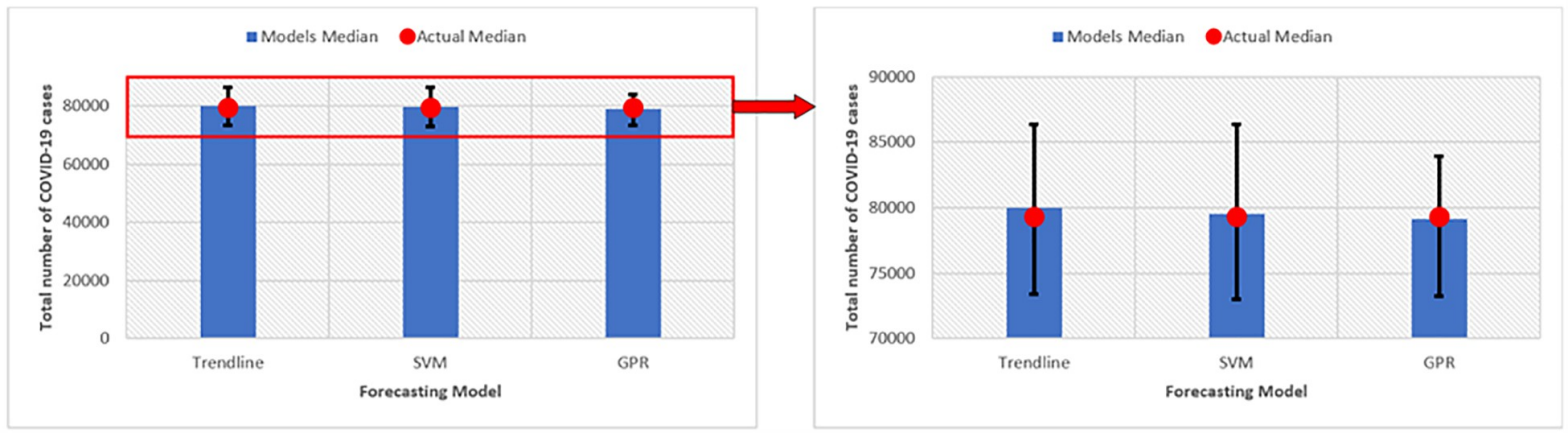
Models uncertainty.

The uncertainty in a prediction model describes the variability in its prediction performance due to application on a plausible dataset (i.e., variability in model output caused from different input values). It is important to quantify a model uncertainty as it reflects its robustness. In this work, the uncertainty in proposed models is assessed by evaluating its variation and deviation from the actual data. For this purpose, the median of each prediction round is calculated and compared to the median of actual data and other prediction models. The deviation of overestimation and underestimation in prediction is calculated through positive error and negative error from the data median. Fig 8 depicts the results of models’ uncertainty. Shown values represent the mean value of all eight rounds.

Fig 8 shows that the eight rounds mean values of the SVM and GPR models median is in closer proximity to the actual median of dataset compared to the Trendline model value. This indicates lower uncertainty in these two models. In addition, investigating the error bar for the three models suggests that the GPR model has the shortest error bar compared to Trendline and SVM models. On average, the error deviation from the median was approximately 5000 in total number of cases compared to 6500 and 6700 for SVR and Trendline, respectively.

## 5. Conclusions

Three short-term forecasting models were proposed to predict the total number of infected COVID-19 cases in Canada. A simple trend analysis model (Trendline), a Support Vector Machine (SVM) regression model, and a Gaussian Processing Regression (GPR) model were employed for this purpose. Forecasting horizons of 5, 10, 15, 20, 25, and 30 days were also investigated in this paper. NRMSE and MAPE were calculated to assess the performance of the forecasting models compared to the actual total number of infected cases. Accordingly, the following remarks can be made:

- GPR and SVM models outperformed the simple trend analysis model (i.e. Trendline) on all forecasting horizons.
- The three proposed models performed better during the later studied months as more information was available with fewer uncertainties and deviations associated with the data.
- 50–70 previous data points were optimal to fit and train the proposed models. Models that were fed with this input data length performed better than other span lengths for the country in question. Such span length was observed here to be long enough to explain important trends. This span length might vary if a different dataset with different trends for a different country was used.
- Although the same models and techniques were used in all forecasting rounds, different functions and Kernels were used to account for the dynamic changes resulting from interventions in Canada. For example, trend analysis models were fit to a cubic polynomial at the beginning of the study period while a quadratic and a quadrable polynomials were a better fit during other rounds.
- The GPR with a Matern 5/2 Kernel outperformed other proposed models in the majority of rounds. The GPR model achieved relatively accurate predictions in majority of the rounds.

The preferred model, the GPR with a Matern 5/2 Kernel, holds promise as a low-cost, relatively simple prediction model which is accessible to municipal governments. In undertaking their own modelling, local governments and health units may be able to tailor both predictions and interventions to their local circumstances.
